# Plasma Heparan Sulfate Structural Variation and Phenotypic Heterogeneity in Pediatric Acute Respiratory Distress Syndrome

**DOI:** 10.21203/rs.3.rs-9337695/v1

**Published:** 2026-05-04

**Authors:** Colin J. Sallee, Aline B. Maddux, Joseph A. Hippensteel, Daniela Markovic, Zhangjie Wang, Jian Liu, Kaori Oshima, Robert P. Richter, Andreas Schwingshackl, Peter M. Mourani, David Elashoff, Eric P. Schmidt, Anil Sapru

**Affiliations:** University of California Los Angeles and Mattel Children’s Hospital; University of Colorado School of Medicine and Children’s Hospital Colorado; University of Colorado Anschutz Medical Campus; University of California Los Angeles; Glycan Therapeutics; University of North Carolina at Chapel Hill; Harvard Medical School and Massachusetts General Hospital; University of Alabama at Birmingham Heersink School of Medicine; University of California Los Angeles and Mattel Children’s Hospital; University of Michigan Medical School; University of California Los Angeles; Harvard Medical School and Massachusetts General Hospital; University of California Los Angeles and Mattel Children’s Hospital

**Keywords:** Pediatric Acute Respiratory Distress Syndrome, Endothelial Glycocalyx, Heparan Sulfate, Heparanase, Biological Heterogeneity, Mass Spectrometry

## Abstract

Endothelial glycocalyx (eGCX) shedding contributes to microvascular endotheliopathy in Acute Respiratory Distress Syndrome (ARDS) and is a potential underrecognized source of phenotypic heterogeneity. In pediatric ARDS (PARDS), we examined whether circulating heparan sulfate (HS) signatures, as readouts of eGCX shedding, capture inter-individual variability beyond other eGCX components and protein biomarkers, whether specific HS structural features are enriched, and whether they correlate with heparanase-1 (HPSE) activity. We retrospectively analyzed plasma samples (2018–2020) from children with and without PARDS. Mass spectrometry quantified glycosaminoglycans and sulfation subtypes alongside HPSE activity, while protein biomarkers were measured by multiplex assay. Among 46 children (36 PARDS, 10 no PARDS), principal component analysis identified three components explaining 63% of variance. The primary component (PC1) was driven by 6-*O*- and *N*-sulfated HS subtypes, while a secondary component (PC2) reflected inflammatory proteins. In PARDS, higher PC1 scores were associated with worse organ dysfunction and fewer ventilator-free days. Higher total HS levels were associated with enrichment of sulfated HS (including 6-*O*- and *N*-sulfated subtypes), whereas the opposite pattern was observed in non-PARDS; higher HPSE activity further correlated with these subtypes. These preliminary findings suggest that variation in circulating HS signatures identifies a distinct endothelial-derived biological axis linked to clinical outcomes.

## INTRODUCTION

Acute respiratory distress syndrome (ARDS) exhibits substantial phenotypic (clinical and biological) heterogeneity that has been postulated to limit the success of therapeutic trials.^1^ Accumulating evidence suggests that the extent of systemic inflammation, reflected in circulating cytokines and innate immune activation markers, partitions patients into prognostic and potentially, predictive biological phenotypes.^2^ Yet, even with the growing use of biological data to support precision medicine strategies in critical care medicine, current ARDS biological phenotyping frameworks remain limited and likely, incomplete.

Microvascular endotheliopathy is a central mechanism of organ injury in ARDS^3^ and critical illness^4^ more broadly; however, its relative contribution to biological heterogeneity and phenotypic classification in ARDS remains comparatively understudied. An early and key driver of microvascular endotheliopathy is shedding of the endothelial glycocalyx (eGCX)^5^, a heparan sulfate (HS) proteoglycan-rich extracellular matrix that lines the luminal surface of the endothelium and is essential for maintaining endothelial barrier integrity^6^, regulating vascular tone^7^, and preserving inflammatory homeostasis.^8^ Disruption of the eGCX by enzymatic “sheddases”^9^, reactive oxygen species^10^, and shear stress^11^ leads to loss of these protective functions. Circulating HS fragments serve not only as mechanistic readouts of eGCX shedding^12,13^ but also display variable sulfation motifs that may actively influence cellular signaling through interactions with protein ligands, growth factors, and cell-surface receptors.^14^ Accordingly, these structural features may provide insight into mechanisms driving eGCX shedding and into their broader contributions to ARDS pathophysiology.

In this study of mechanically ventilated children with pediatric ARDS (PARDS), we pursued three complementary objectives: (1) to examine whether circulating signatures of eGCX-derived HS detects biological variability distinct from other putative eGCX components and canonical ARDS protein biomarkers; (2) to assess whether specific HS structural features (i.e., sulfation motifs), are selectively enriched in PARDS; and (3) in an exploratory analysis, to evaluate the relationship between HS signatures and heparanase-1 (HPSE) activity, the principal HS degrading enzyme.^15^ We hypothesized that circulating eGCX-derived HS signatures capture biological heterogeneity in children with PARDS that is not reflected by other eGCX components or conventional protein biomarkers. We further hypothesized that variation in HS signatures arises not only from differences in overall abundance but also from qualitative changes in sulfation patterns.

## METHODS

### Study Design:

The parent study was conducted from 2018–2020 at an urban, quaternary children’s hospital.^16^ It was approved by the Colorado Multiple Institutional Review Board (IRB 18-0463) and conducted in accordance with institutional standards and the 1975 Helsinki Declaration. Eligible patients’ parent/guardians were approached to obtain written informed consent for participation.

We conducted a retrospective, secondary analysis of this prospectively enrolled cohort of children (≥ 1 month and ≤ 18 years old) with acute respiratory failure (ARF) requiring invasive mechanical ventilation (IMV) for a minimum of three days. The original study excluded patients who had support limitations, if the investigator or PICU team felt the child was inappropriate to enroll, or if the patient had a pre-existing tracheostomy. Blood was collected on day of consent, which was typically 3–5 days after IMV initiation. Among patients with available blood for analysis, a convenience sample of children with and without PARDS was identified from the parent cohort. PARDS diagnosis was based on the Pediatric Acute Lung Injury Consensus Conference-2 (PALICC-2) criteria.^17^ This group was compared to children with ARF receiving IMV for neurologic failure or a non-pulmonary procedure/surgery similar to prior analyses of this cohort.^18^ These patients without PARDS served as a critically ill comparator group whose disease processes were not suggestive of acute lung injury at enrollment.

The present study used a de-identified dataset and blood samples for analysis and was deemed IRB exempt (UCLA IRB #22-001375). Plasma was analyzed from the first collection after enrollment in the study.

### Definitions:

We collected demographics (age and sex), comorbidities (presence of chronic pulmonary conditions, neurologic disease, immunocompromise [including oncologic disease or hematopoietic cell transplantation]), elements specific to PARDS (risk factors, chest imaging, and oxygen saturations), illness severity (pediatric risk of mortality score [PRISM III]).

Vital sign, ventilator, and laboratory data were used to calculate organ dysfunction indices (oxygen saturation index [OSI]; pediatric logistic organ dysfunction score[PELOD]-2). Daily OSI^19^ (index of pulmonary dysfunction) and PELOD-2^20^ (index of multiple organ dysfunction) were calculated using the most abnormal values from each calendar day. As a sensitivity analysis, we excluded the respiratory component of the PELOD-2 scoring system to derive the non-pulmonary PELOD-2 score. Data collection focused on the first week of IMV (day 0 = day of intubation) with organ dysfunction scores recorded once daily up to 7 days unless the patient died or was discharged from the PICU (whichever occurred first). We calculated maximum organ dysfunction and cumulative (∑) organ dysfunction scores during this timeframe for use in regression analyses. Given that plasma collection could have been contemporaneous to (or occurred after) maximum organ dysfunction, cumulative organ dysfunction scores were computed to preserve exposure-outcome temporal alignment and provide a more complete measure of organ dysfunction burden.

Clinically relevant outcomes included 28-day ventilator free-days and PICU-free days. Ventilator-free and PICU-free days were determined by subtracting total ventilator or PICU days from 28 in survivors. Patients with ≥ 28 days of IMV, PICU admission, and non-survivors were assigned a value of 0.

### Molecular Measurements:

#### Glycosaminoglycans (GAGs):

**Figure 1:**
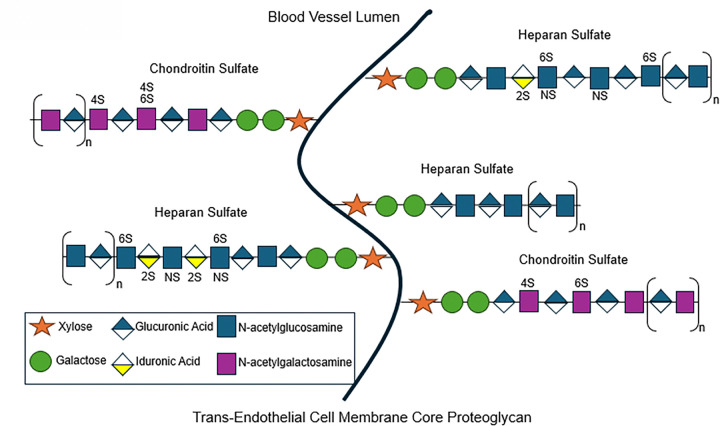
The endothelial glycocalyx. The main constituents include proteoglycans (e.g., syndecan-1) and glycosaminoglycans (GAGs), such as heparan sulfate (HS), chondroitin sulfate (CS), and hyaluronic acid (HA). GAGs are linear polysaccharides composed of repeating disaccharide units consisting of a hexuronic acid (glucuronic acid [GlcA] or iduronic acid [IdoA]) linked to a hexosamine (N-acetylglucosamine [GlcNAc] or galactosamine [GalNAc]). HS and CS can be further modified by sulfation of their disaccharide units. HS, in particular, exhibits staggering structural complexity due to variable sulfation at the 6-*O*- (“6S”) and/or amino (“NS”) positions of GlcNAc, as well as the 2-*O*- (“2S”) position of IdoA. This pattern of sulfation along the HS chain generates a heterogeneous distribution of negative charge, enabling highly specific interactions with positively charged regions of soluble and cell-surface proteins. In contrast, HA (not pictured) is non-sulfated and, unlike other GAGs, is not covalently attached to the endothelial cell surface via proteoglycans. Instead, it associates non-covalently with cell-surface glycoproteins (not pictured). Note that sulfation at the 3-*O*- (“3S”) position of the GlcNAc residue of HS may also exist within glycocalyx HS, but glycoanalytical techniques at the time of analysis were insensitive to detection of this motif.

High performance liquid chromatography tandem mass spectrometry (HPLC-MS/MS) was previously performed on plasma samples to quantify (ng/mL) GAGs including HS, chondroitin sulfate (CS), and hyaluronic acid (HA), and characterize HS and CS disaccharide sulfation subtypes. Plasma levels of sulfated and non-sulfated HS (glucuronic or iduronic acid, or ΔUA, linked to N-acetylglucosamine, or GlcNAc) and CS (glucuronic acid, or ΔUA, linked to N-acetylgalactosamine, or GalNAc) disaccharides were measured after enzymatic digestion of total (or unfractionated) HS and CS. Unlike HS and CS, HA (glucuronic acid, or ΔUA, linked to GlcNAc) is neither sulfated nor covalently bound to the endothelial cell membrane. Additional details regarding the state-of-the-art HPLC-MS/MS methodology^13,21,22^ are available in the e-supplement. Visual representation of the eGCX, including its GAG constituents and associated sulfation motifs, is shown in Fig. 1.

#### Proteins and Proteoglycans:

Protein biomarkers associated with innate immune and endothelial activation and lung epithelial injury were measured in plasma alongside proteoglycans, endocan and syndecan-1 (a main HS proteoglycan), using two magnetic bead multiplex panels (Luminex, R&D Systems, Minneapolis, MN): a 5-plex high performance, high sensitivity human cytokine panel and a 11-plex human discovery assay (e-supplement).

### HPSE Activity:

HPSE activity (U/mL) was measured in plasma using our novel HPLC-MS/MS-based method. In this method, the chemically synthesized dodecasaccharide, known as dekaparin, is utilized as a substrate, which, upon cleavage by HPSE, releases a specific disaccharide with a structure of GlcNS6S-GlcA (glucuronic acid) and then measured via HPLC-MS/MS (e-supplement).

### Statistical Analysis:

Full methodological details are provided in the e-supplement, with all studied biomarkers listed in e-Table 1. The primary analyses are summarized below.

Principal component analysis (PCA) was used as an unsupervised method to identify latent sources of biological variation in the dataset. Retained principal components (PC) were interpreted as candidate biological axes. PCA was performed on the correlation matrix of GAG, protein, and proteoglycan markers using the prcomp function (R version 4.2.1, R Foundation for Statistical Computing, Vienna, Austria). Each PC represented a weighted linear combination of measured biomarkers. Loading coefficients indicated the magnitude and direction of each biomarker’s contribution to the variance explained by the PC; biomarkers with larger absolute loadings contributed more strongly. Loadings were visualized using heat maps, and PCs were visualized using biplots. Retained PCs were evaluated for associations with organ dysfunction and clinical outcomes in PARDS. For each patient, PC scores (unitless values reflecting alignment between an individual’s biomarker profile and each PC) were calculated and used as explanatory variables in multivariable linear regression models. Standardized regression coefficients (β) with 95% confidence intervals (CIs) were estimated, where β represented the change in the outcome (in standard deviation [SD] units) associated with a 1 SD increase in PC score.

We next examined whether structural features of HS, specifically sulfated versus non-sulfated motifs (HS-0S), were selectively enriched in children with and without PARDS. A composite sulfated HS metric was calculated by summing concentrations of all sulfated HS disaccharide subtypes. The proportion of sulfated HS was defined as sulfated HS (ng/mL) divided by total (or unfractionated) HS (ng/mL). Linear regression models were used to assess whether the proportion of sulfated HS varied with total HS levels. Effect modification by PARDS status was evaluated by including an interaction term (proportion sulfated HS ~ log total HS * PARDS status). Differences in regression slopes (Δ slope) between groups were estimated with 95% CIs. All analyses were repeated for CS.

Exploratory analyses assessed associations between HPSE activity and circulating HS and its subtypes using Spearman correlation (ρ) and regression models. These analyses were considered hypothesis-generating.

In a prior study^23^, we identified three distinct PARDS patient clusters (Cluster 1, n = 12; Cluster 2, n = 19; Cluster 3, n = 5) characterized by stepwise increases in absolute concentrations of HS disaccharide subtypes. Cluster 3 identified a severe eGCX shedding phenotype associated with higher disease severity and worse clinical outcomes. We examined whether the relative proportion of sulfated HS and HPSE activity differed across these previously defined patient clusters.

Finally, given the small sample size, prespecified sensitivity analyses accounting for potential influential observations were performed to assess the robustness and internal validity of the findings (e-supplement).

## RESULTS

### Patient Characteristics:

This study included 46 children: 36 children with PARDS and 10 without PARDS. Demographic characteristics, clinical variables, illness severity were similar between groups (e-Table 2). Plasma collection occurred a similar number of days after initiation of IMV in patients with and without PARDS (median 4.6 days; interquartile range [IQR] 3.4–5.6 vs. 4.3; IQR 3.1–5.3, respectively). OSI was higher in children with PARDS (8.3; IQR 5.4–13 vs. 1.9; IQR 1.8–4.4).

### Principal Component Analysis:

**Figure 2:**
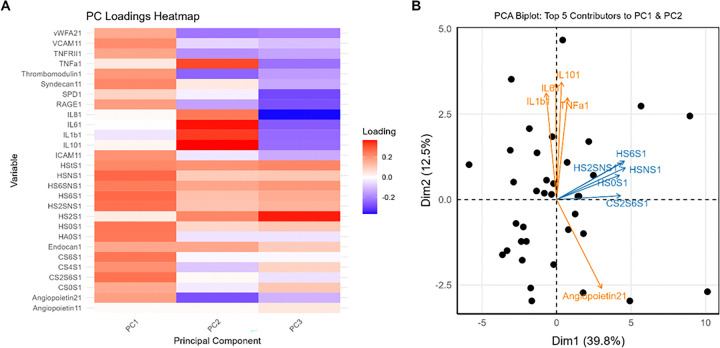
PC Loadings Heatmap. **(A)** Each PC summarizes the main axes of biological variation in the dataset and represents a weighted linear combination of 28 variables listed. Variables with larger loadings (absolute value of the loading coefficient) contribute more to the variability detected by a given PC. Variables with positive loadings indicate that variable positively correlated with a given PC. **(B) PCA Biplot of PC1 and PC2.** The blue and orange arrows indicate the top 5 biomarker contributors (highest absolute value of loading coefficients) to PC1 (Dim 1=”glycocalyx axis”) and PC2 (Dim 2, “inflammatory axis”), respectively. The length of each arrow reflects the strength of that variable’s contribution to a given PC, with longer arrows indicating greater importance. Arrows pointing toward the positive side of a PC indicate a positive correlation with that PC, meaning that higher PC values correspond to higher levels of that variable. The scatter plot shows the relationships between patient (data points) and the principal components. The position of each point in the 2-dimensional plane indicates the values of PC1 and PC2 for a given patient. PCA = principal component analysis; PC = principal component.

In children with PARDS, PC1-PC3 were retained based on scree plots and permutation testing (e-Figure 1), together explaining 63% of the total variance (PC1: 40%, PC2: 12%, PC3: 11%; all p < 0.0001). PC1 was primarily defined by eGCX-derived HS, with HS subtypes modified with 6-*O*- and *N*-sulfation and the non-sulfated (HS-0S) subtype exhibiting the largest positive loading coefficients. In contrast, PC2 reflected an inflammatory signature influenced by interleukin (IL)-1β, -6, -10, and TNF-α (Fig. 2). After exclusion of identified influential observations which included two patients from Cluster 3 (e-Figure 2A), HS disaccharide subtypes modified with 6-*O*- and *N*-sulfation remained the dominant contributors to PC1 (e-Figure 2B).

Among children with PARDS, higher PC1 scores (reflecting patients with higher values of positive loading variables on PC1, which in this case, indicated more shedding of eGCX-derived HS) were associated with higher ∑OSI, ∑PELOD-2, and ∑non-pulmonary PELOD-2 after adjustment for PC2 and PC3 (Table 1). An inverse association between PC1 and 28-day ventilator-free days was observed after adjustment for PC2 and PC3 (e-Table 3).

**Figure 3:**
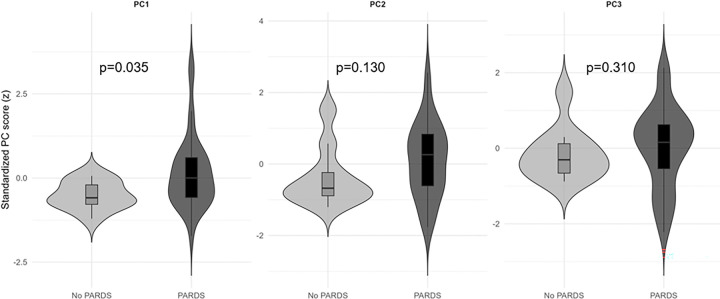
Standardized PC scores by PARDS status. PC1 (“glycocalyx axis”) scores were significantly higher in children with PARDS relative to those without PARDS. p<0.05 was statistically significant. PC = principal component; PARDS = pediatric acute respiratory distress syndrome.

Incorporation of children without PARDS into the PCA yielded similar PC axes with visual separation of PARDS and non-PARDS patients along the PC1 and PC2 axes (e-Figure 3). PC1 scores were significantly higher in children with compared to without PARDS (p = 0.035, Fig. 3).

### Sulfation Patterns of GAGs in Children with and without PARDS:

The relative proportion of sulfated HS subtypes, although higher in children with PARDS, did not significantly differ from non-PARDS (0.21; IQR 0.13, 0.29 vs. 0.15; IQR 0.11, 0.21; p = 0.130).

**Figure 4:**
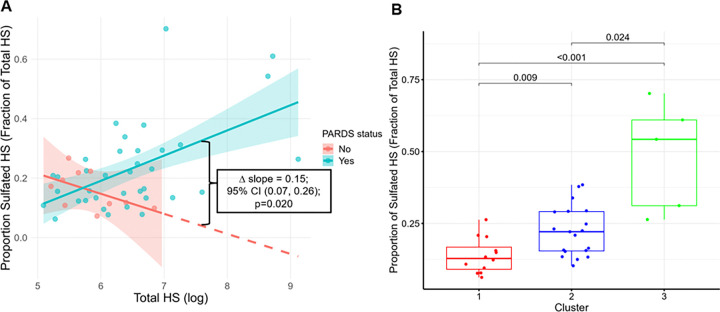
**(A) The proportion of sulfated HS as a function of total (or unfractionated) HS in children with and without PARDS.** In pediatric ARDS, higher total HS levels were associated with a higher proportion of sulfated HS (β per log-HS increase=0.08; 95% CI 0.03, 0.13). In contrast, among ventilated children without PARDS (i.e., those requiring invasive mechanical ventilation but without lung injury or sepsis at enrollment), higher total HS levels were associated with a lower proportion of sulfated HS (β per log-HS increase = −0.06; 95% CI, −0.15, −0.02). A linear model including the interaction term: Proportion Sulfated HS ~ Total HS * PARDS status demonstrated that the slopes differed significantly between groups. p<0.05 was statistically significant. **(B) The proportion of sulfated HS compared to previously identified PARDS clusters.** Each cluster was previously characterized by increasing absolute levels of all HS subtypes (both sulfated and non-sulfated HS). Cluster 3 was characterized by the highest levels of shedding of eGCX-derived HS and was further associated with greater PARDS severity and worse clinical outcomes. In this figure, the proportion of sulfated HS (all sulfated HS subtypes as a fraction of total HS) increased across clusters (Kruskal-Wallis p<0.001), indicating heterogeneity in circulating HS signatures is both quantitative and qualitative, with enrichment of sulfated HS motifs across clusters. Post-hoc Dunn tests identified significant inter-cluster differences (all pairwise p<0.05). p<0.05 was considered statistically significant. HS = heparan sulfate; PARDS = pediatric acute respiratory distress syndrome.

However, when the proportion of sulfated HS was modeled as a function of total (or unfractionated) HS, these relationships significantly differed by PARDS status (p = 0.020, Fig. 4A). In children with PARDS, the proportion of sulfated HS increased with total HS levels (β per log-HS increase = 0.08; 95% CI: 0.03, 0.13). In contrast, among children without PARDS, increasing total HS levels were associated with a lower proportion of sulfated HS (β per log-HS increase= −0.06; 95% CI: −0.15, −0.02). The relative proportion of sulfated HS differed significantly across PARDS clusters (Kruskal-Wallis p < 0.001), with Cluster 3 demonstrating the highest relative proportion of sulfated HS (all pairwise p < 0.05; Fig. 4B).

The relationship between total HS levels and the proportion of HS disaccharide subtypes expressing 6-*O*- and *N*-sulfation (subtypes that loaded strongly on PC1) also varied by PARDS status (Δ slope = 0.11; 95% CI, 0.05, 0.19; p = 0.012, e-Figure 4A) In addition, the relative proportion of HS expressing these sulfation subtypes differed between PARDS clusters (Kruskal-Wallis p < 0.001), with the relative proportion of 6-*O*- and *N*-sulfated enriched HS increasing stepwise across clusters (all pairwise p < 0.05, e-Figure 4B).

The proportion of sulfated CS increased with total CS levels among children with PARDS, but there was not strong evidence these relationships varied by PARDS status (data not shown). Sensitivity analyses excluding the same influential observations from Cluster 3 in the PCA models yielded similar effect estimates for all analyses.

### HPSE Activity:

HPSE activity was higher in children with PARDS compared to non-PARDS, although this difference did not reach statistical significance (e-Figure 5). Children with PARDS exhibited a 10-fold greater variance in HPSE activity relative to non-PARDS (Brown-Forsythe test, p = 0.002).

**Figure 5:**
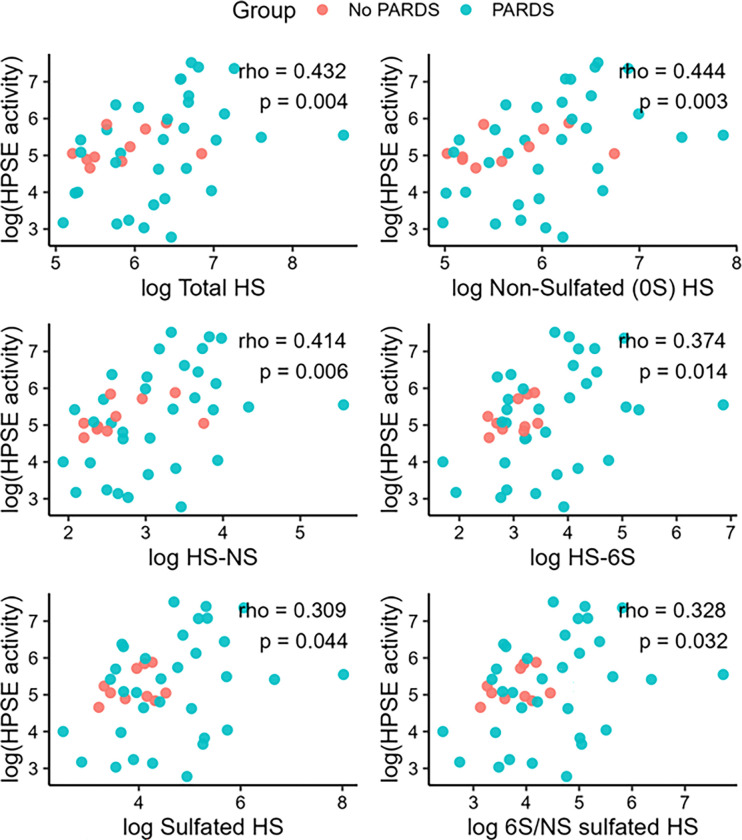
Correlations between HPSE activity and total (unfractionated) HS and HS disaccharide subtypes. Sulfated HS was the summation of all sulfated HS disaccharide subtypes. 6S/NS HS represented the summation of all subtypes enriched with 6-*O*- and *N*-sulfation. p<0.05 was statistically significant. HPSE = heparanase-1; HS = heparan sulfate.

Higher HPSE activity demonstrated a modest positive correlation with total HS levels (Spearman ρ = 0.28, p = 0.054). Regression diagnostics identified the same two patients from Cluster 3 as influential observations (e-supplement). These patients exhibited the highest total HS levels but among the lowest HPSE activity levels. After excluding these observations, the correlation between HPSE activity and total HS strengthened (ρ = 0.43, p = 0.004). HPSE activity also positively correlated with specific HS disaccharide subtypes, including HS-0S, HS-NS, and HS-6S. Significant correlations were observed when all sulfated subtypes were analyzed collectively, as well as when 6-*O*- and *N*-sulfated HS subtypes were examined in aggregate (Fig. 5).

**Figure 6:**
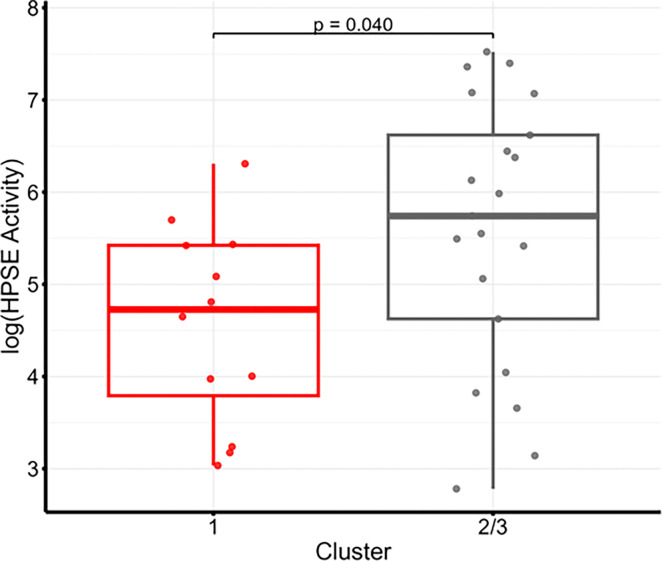
HPSE activity stratified by PARDS clusters. Cluster 1 (n=12) compared to combined Cluster 2 and 3 (n=22) after removal of statistically defined outliers (n=2) from Cluster 3. HPSE activity was significantly higher in children in Cluster 2/3 relative to Cluster 1. p<0.05 was statistically significant. PARDS = pediatric acute respiratory distress syndrome; HPSE = heparanase-1.

Following exclusion of the two influential observations from Cluster 3 and merging Clusters 2 and 3 (due to small sample size), HPSE activity was higher in the combined group (n = 22) compared with Cluster 1 (p = 0.040, Fig. 6).

## DISCUSSION

In this study of children of mechanically ventilated children with PARDS, we found that (1) shedding of eGCX-derived HS into the circulation was a major and clinically significant contributor to inter-individual biological variation; (2) higher HS abundance was associated with selective enrichment of its sulfated motifs and; (3) in exploratory analyses, higher HPSE-1 activity positively correlated with the abundance of HS in circulation.

Our prior investigations demonstrated that circulating total (or unfractionated) HS levels were elevated in children with PARDS compared with those without PARDS.^18^ Furthermore, circulating HS profiles were also heterogeneous in PARDS, and children with differentially elevated levels of sulfated *and* non-sulfated HS disaccharide subtypes were more severely ill and experienced worse clinical outcomes.^23^ Building on this work, our current findings suggest that release of HS fragments into the circulation (as mechanistic readouts of eGCX shedding) appears to define a unique topography within the complex biological landscape of PARDS. HS subtypes, specifically those modified with 6-*O*- and *N*-sulfation, contributed more strongly to the variance along the primary biological axis (PC1) than other putative eGCX components including CS and HA. Although CS is not a specific marker of eGCX shedding, as it also circulates as part of bikunin within inter-alpha-inhibitor^24^, the biological significance of CS-2S6S, an important contributor to PC1, remains unclear and warrants further investigation.

These findings also seem to suggest that shedding of eGCX-derived HS represents a distinct and coordinated biological axis rather than a non-specific epiphenomenon of systemic inflammation or generalized endothelial activation. Studies in adult and pediatric ARDS have demonstrated that the degree of inflammation, characterized by canonical circulating cytokines and markers of innate immune activation, partitions patients into clinically meaningful subphenotypes.^25,26^ In contrast, conventional protein biomarkers of endothelial cell activation, although meaningfully altered, have not best distinguished the hyperinflammatory state^27^ or enabled comparable biological subphenotyping. ARDS fundamentally involves disruption of the lung-vascular interface^28^, and the extent and mechanisms of endotheliopathy are also unlikely to be homogeneous across patients. Robust preclinical data implicate eGCX shedding as an early contributor to the endotheliopathy characteristic of ARDS pathogenesis.^29–31^ As such, circulating HS signatures may have the mechanistic specificity, temporal resolution, and dimensional depth to identify novel subgroups characterized by distinct endothelial pathobiology.

Although eGCX shedding and innate immune activation were shown to be statistically orthogonal, they are likely to be mutually reinforcing *in vivo*. HS is crucial to establishing chemokine gradients necessary for the directional migration of leukocytes, acting as a ligand for leukocyte selectins, and sequestering cytokines and growth factors, protecting them from enzymatic cleavage.^8^ Soluble HS fragments can also signal through MyD88-dependent pathways, including TLR4, leading to NF-κB activation and downstream cytokine release^32^, while inflammatory cytokines, including TNF-α, IL-1β, IL-6, IL-8, and IL-10 reciprocally activate upstream sheddases that contribute to eGCX shedding.^33^ Within this framework, innate immune activation and eGCX shedding represent intersecting but non-identical biological axes rather than interchangeable manifestations of the same pathological process. In some patients, convergence of these axes may define a high-risk subphenotype with potential prognostic and therapeutic implications.

Our findings further suggest that heterogeneity in circulating HS signatures in PARDS reflects not only quantitative differences in HS levels but also qualitative or compositional shifts in HS structure (i.e., sulfation motifs). We show that under baseline conditions, circulating HS is largely a low sulfation species,^34^ and that group-related differences in HS abundance were accompanied by changes in HS sulfation features. In children with PARDS, increasing total circulating HS levels were accompanied by disproportionate enrichment of sulfated HS motifs, including those with modified with 6-*O*- and *N*-sulfation. In contrast, among children without PARDS, non-sulfated species predominated, a profile perhaps more consistent with non-pathologic or non-enzymatic turnover of the eGCX. Collectively, these findings suggest that measuring HS abundance alone does not fully capture the complexity of eGCX shedding. Instead, enrichment of specific HS sulfation motifs suggests that HS structure encodes mechanistically and clinically relevant information, while also serving as a potential biomarker of disease severity in PARDS.

These novel observations are important as preclinical evidence suggests that the HS fragments released from the eGCX are not biologically inert. Rather, fragments of sufficient size and sulfation can influence pathologic cellular signaling through paracrine, autocrine, and endocrine mechanisms.^35^ These sulfated HS fragments may function as endogenous danger signals^32,36^, amplifying inflammatory and endothelial signaling, sequestering antimicrobial peptides^37,38^, and further disrupting tissue homeostasis in a sulfation-dependent manner.^12^ In a small adult study, patients with indirect lung injury were found to have circulating HS fragments approximately 6–8 saccharides in length that were enriched with sulfation, postulated to result from HPSE-mediated cleavage.^39^ HPSE is the only known mammalian endoglycosidase capable of directly cleaving glycocalyx HS. Beyond its direct enzymatic activity on the eGCX, HPSE may modulate eGCX damage indirectly and through non-enzymatic mechanisms.^40^ Accordingly, defining the substrate specificity of HPSE and the HS fragments it generates has attracted considerable attention, as these pathways may reveal novel therapeutic targets in critical illness where microvascular endotheliopathy is a common pathway of disease pathogenesis.

Our study provides proof of concept that in PARDS, shedding of eGCX-derived HS into the circulation is shaped, in part, by the enzymatically active HPSE. Using a novel functional HPSE assay, we linked the circulating abundance of HS to a defined upstream mediator of eGCX shedding. Preclinical work suggests that HPSE may preferentially target highly sulfated regions of HS by hydrolyzing internal GlcA(β1–>4)GlcNAc linkages^41,42^, generating short, highly sulfated, bioactive fragments.^43^ We observed correlations between HPSE activity and specific HS disaccharide subtypes, including 6-*O*- and *N*-sulfated species. While it may be tempting to attribute the enrichment of 6-*O*- and *N*-sulfated HS subtypes in PARDS solely to HPSE-mediated cleavage, HS sulfation is regulated by multiple, interconnected mechanisms. These include 6-*O*-sulfatase activity,^34^ changes in systemic clearance,^44^ and lysosomal processing of HS fragments,^45^ all of which may be altered in critical illness. Accordingly, these observations, while novel, should be interpreted as exploratory and hypothesis-generating.

Our findings raise other important biological considerations. The two statistically aberrant observations in Cluster 3, characterized by the highest circulating HS burden (particularly sulfation-enriched HS), paradoxically exhibited among the lowest measurable HPSE activity, suggesting that more complex regulatory mechanisms may be at play. The relationship between HPSE activity and eGCX shedding may be non-linear, such that eGCX shedding reaches a critical threshold at which substrate availability (i.e., cleavable HS on the endothelial surface) becomes limiting, resulting in lower measurable HPSE activity. Alternatively, regulatory feedback mechanisms, possibly mediated by HS fragments themselves, may suppress HPSE activity.^46^ Taken together, these findings underscore the need to clarify how HPSE and HS are temporally associated and their relative contributions to organ dysfunction and clinically relevant outcomes.

A few limitations merit consideration. First, this study included a small cohort with cross-sectional biological sampling, which limits inference regarding temporal dynamics and causality. The timing of plasma collection is particularly important in ARDS, where the precise onset of microvascular endotheliopathy (or any meaningful pathobiology) is difficult to ascertain. Although sampling beyond three days after initiation of IMV was restricted by the original study design, prior research suggests that biomarkers measured several days after clinically recognized illness onset may provide greater prognostic value.^47^ Therefore, these findings require validation in larger, independent cohorts that are adequately powered to evaluate temporal trajectories and associations with clinical outcomes. Second, eGCX shedding may also occur through additional mechanisms, including matrix metalloproteinase (MMP)-mediated cleavage of proteoglycan core proteins such as syndecan-1.^48^ HPSE-mediated shedding of HS from syndecan-1 exposes its ectodomain, making it more susceptible to proteolytic cleavage. In addition, HPSE can simultaneously upregulate MMP activity, indirectly enhancing eGCX shedding.^49^ These interactions could not be assessed in the present study and warrant further investigation. Third, although our glycomic analytical platform uses a state-of-the-art technique, it has inherent limitations in spatial resolution. While we provide detailed characterization of circulating HS abundance and sulfation patterns, we are unable to localize shedding to specific organs or vascular beds. Additionally, we cannot fully determine HS chain length with comprehensive sulfation mapping, which could enable identification of specific fragments generated by HPSE mediated shedding, and lend insights into microvascular segment- and organ-specific injury.

## CONCLUSIONS

These findings suggest that eGCX-derived HS shedding identifies a distinct endothelial-derived biological axis in PARDS, with patterned enrichment of sulfated HS fragments that may carry mechanistic and clinical relevance. Although validation in larger cohorts is needed, these results highlight the potential of eGCX shedding and its regulatory pathways to inform biological phenotyping in PARDS and improve risk stratification and targeted therapeutic strategies.

## Supplementary Material

Supplementary Files

This is a list of supplementary files associated with this preprint. Click to download.

• CSalleeSupplement46.docx

## Figures and Tables

**Table 1:** Association between PCs and max and cumulative organ dysfunction indices.

Principal Component	Max OSI β (95% CI)	ΣOSI β (95% CI)
PC1	0.13 (95% CI −0.20, 0.46), p = 0.437	0.52 (95% CI 0.22, 0.81), **p = 0.001**
PC2	0.25 (95% CI −0.08, 0.59), p = 0.133	0.26 (95% CI −0.26, 0.32), p = 0.855
PC3	0.19 (95% CI −0.14, 0.53), p = 0.246	−0.23 (95% CI −0.52, 0.06), p = 0.119
	Max PELOD-2	ΣPELOD-2
PC1	0.34 (95% CI 0.02, 0.66), **p = 0.035**	0.52 (95% CI 0.22, 0.81), **p = 0.001**
PC2	−0.11 (95% CI −0.42, 0.21), p = 0.497	0.36 (95% CI −0.25, 0.33), p = 0.802
PC3	−0.29 (95% CI −0.61, 0.02), p = 0.066	−0.23 (95% CI −0.53, 0.06), p = 0.111
	Max Non-Pulmonary PELOD-2	ΣNon-Pulmonary PELOD-2
PC1	0.29 (95% CI −0.02, 0.62), p = 0.071	0.57 (95% CI 0.29, 0.85), **p < 0.001**
PC2	−0.62 (95% CI −0.38, 0.26), p = 0.698	0.10 (95% CI −0.17, 0.38), p = 0.444
PC3	−0.30 (95% CI −0.62, 0.02), p = 0.065	−0.25 (95% CI −0.53, 0.02), p = 0.070

Cumulative (Σ) organ dysfunction scores were calculated as summative indices up to the first week after initiation of invasive mechanical ventilation. Standardized regression coefficients (β) with 95% confidence intervals (CIs), with β representing the change in the outcome variable in standard deviation units [SD]) associated with a 1 SD change in PC score. p < 0.05 was statistically significant. Non-pulmonary PELOD has respiratory component removed. OSI = oxygen saturation index; PC = principal component; PELOD = pediatric logistic organ dysfunction.

## Data Availability

The datasets used and/or analyzed during the current study are available from the corresponding author on reasonable request.

## Abbreviations

ARDS: Acute Respiratory Distress Syndrome
eGCX: endothelial glycocalyx
HS: heparan sulfate
PARDS: pediatric ARDS
HPSE: heparanase-1
ARF: acute respiratory failure
IMV: invasive mechanical ventilation
PALICC-2: Pediatric Acute Lung Injury Consensus Conference-2
PRISM III: pediatric risk of mortality score
OSI: oxygen saturation index
PELOD: pediatric logistic organ dysfunction score
GAG: glycosaminoglycan
HPLC-MS/MS: High performance liquid chromatography tandem mass spectrometry
CS: chondroitin sulfate
HA: hyaluronic acid
GlcNAc: N-acetylglucosamine
GalNAc: N-acetylgalactosamine
GlcA: glucuronic acid
IdoA: iduronic acid
PCA: principal component analysis
PC: principal component
CI: confidence interval
SD: standard deviation
IQR: interquartile range
